# Complete genome sequences and genomic characterization of five plasmids harbored by environmentally persistent *Cronobacter sakazakii* strains ST83 H322 and ST64 GK1025B obtained from powdered infant formula manufacturing facilities

**DOI:** 10.1186/s13099-022-00500-5

**Published:** 2022-06-06

**Authors:** Flavia J. Negrete, Katie Ko, Hyein Jang, Maria Hoffmann, Angelika Lehner, Roger Stephan, Séamus Fanning, Ben D. Tall, Gopal R. Gopinath

**Affiliations:** 1 Center for Food Safety and Applied Nutrition, Office of Applied Research and Safety Assessment, U. S. Food and Drug Administration, Laurel, MD USA; 2 Joint Institute for Food Safety and Applied Nutrition, University of Maryland College Park, College Park, MD USA; 3 Center for Food Safety and Applied Nutrition, Office of Regulatory Science, Food and Drug Administration, College Park, MD USA; 4 Institute for Food Safety and Hygiene, University of Zürich, Zurich, Switzerland; 5 WHO Collaborating Centre for Cronobacter, University College Dublin, Dublin, Ireland; 6 School of Public Health, Physiotherapy and Population Science, UCD Centre for Food Safety, University College Dublin, Dublin, Ireland

**Keywords:** *Cronobacter sakazakii*, Whole genome sequencing, Plasmids, Built environment, Complete genomes, PHASTER, Phage-plasmids

## Abstract

**Background:**

*Cronobacter sakazakii* is a foodborne pathogen that causes septicemia, meningitis, and necrotizing enterocolitis in neonates and infants. The current research details the full genome sequences of two extremely persistent *C. sakazakii* strains (H322 and GK1025B) isolated from powdered infant formula (PIF) manufacturing settings. In addition, the genetic attributes associated with five plasmids, pH322_1, pH322_2, pGK1025B_1, pGK1025B_2, and pGK1025B_3 are described.

**Materials and Methods:**

Using PacBio single-molecule real-time (SMRT^®^) sequencing technology, whole genome sequence (WGS) assemblies of *C. sakazakii* H322 [Sequence type (ST)83, clonal complex [CC] 83) and GK1025B (ST64, CC64) were generated. Plasmids, also sequenced, were aligned with phylogenetically related episomes to determine, and identify conserved and missing genomic regions.

**Results:**

A truncated ~ 13 Kbp type 6 secretion system (T6SS) gene cluster harbored on virulence plasmids pH322_2 and pGK1025B_2, and a second large deletion (~ 6 Kbp) on pH322_2, which included genes for a tyrosine-type recombinase/integrase, a hypothetical protein, and a phospholipase D was identified. Within the T6SS of pH322_2 and pGK1025B_2, an arsenic resistance operon was identified which is in common with that of plasmids pSP291_1 and pESA3. In addition, PHASTER analysis identified an intact 96.9 Kbp *Salmonella* SSU5 prophage gene cluster in pH322_1 and pGK1025B_1 and showed that these two plasmids were phylogenetically related to *C. sakazakii* plasmids: pCS1, pCsa767a, pCsaC757b, pCsaC105731a. Plasmid pGK1025B_3 was identified as a novel conjugative *Cronobacter* plasmid. Furthermore, WGS analysis identified a ~ 16.4 Kbp type 4 secretion system gene cluster harbored on pGK1025B_3, which contained a phospholipase D gene, a key virulence factor in several host–pathogen diseases.

**Conclusion:**

These data provide high resolution information on *C. sakazakii* genomes and emphasizes the need for furthering surveillance studies to link genotype to phenotype of strains from previous investigations. These results provide baseline data necessary for future in-depth investigations of *C. sakazakii* that colonize PIF manufacturing facility settings and genomic analyses of these two *C. sakazakii* strains and five associated plasmids will contribute to a better understanding of this pathogen's survival and persistence within various “built environments” like PIF manufacturing facilities.

**Supplementary Information:**

The online version contains supplementary material available at 10.1186/s13099-022-00500-5.

## Background

*Cronobacter sakazakii* is an opportunistic foodborne pathogen that causes serious intestinal and extraintestinal systemic infections such as acute gastroenteritis, septicemia, meningitis, urosepsis, osteomyelitis, wound infections, and pneumonia in individuals of all ages [1–5]. Pre-term, low-birth weight, and/or immune compromised neonates and infants are highly susceptible to *C. sakazakii*. Moreover*,* severe invasive infections such as septicemia, meningitis, and necrotizing enterocolitis are hallmarks of this organism’s pathogenicity. Additionally, outcomes from such invasive infantile infections often leave individuals with lifelong debilitating and neurologic impairments such as developmental delays, hydrocephaly, mental retardation, and other chronic neurological sequelae [3, 6, 7]. *C. sakazakii* infections observed in these individuals have been epidemiologically linked to consumption of intrinsically and extrinsically contaminated lots of reconstituted powdered infant (PIF) and follow up formulas; thus, contamination of such products is a challenging task for both infant formula manufacturers and caretakers [7–10]. Another trend that both clinicians and public health scientists must recognize is that unsafe personal hygiene breast-feeding practices, such as the use of contaminated personalized breast pumps, may also lead to infantile infections such as septicemia and meningitis [11–14].

Chase et al*.* [15] described *C. sakazakii* H322, as a highly persistent sequence type (ST) 83, clonal complex (CC) 83 strain that was obtained from a lot of contaminated PIF manufactured in Europe that was never released to the public. Chase et al*.* [15] further showed that the persistence of *C. sakazakii* H322 and other phylogenetically related ST83 strains, which were also found within the production environment of this facility, and its presence could be traced back for more than four years. Microarray analysis showed that these strains differed among them by sequence divergence in 5–38 genes [15]. In separate studies, Gopinath et al. [16] and Chase et al. [29] described several malonate-positive ST64, CC64 *C. sakazakii* strains, including GK1025B (a PIF manufacturing environmental isolate), that were found persisting in the environments of another European PIF manufacturing facility. These ST64 strains were phylogenetically related to other strains obtained from sources such as clinical samples, environments of USA dairy powder manufacturing facilities, spices, and mushrooms from the Middle East and China. Draft whole genome sequence (WGS) assemblies of these strains, together with other PIF production environmental-associated strains, confirmed a ST phylogenetic relatedness among them [16]. In the present study, we report the completed genome sequences of these two highly persistent *C. sakazakii* strains, H322 and GK1025B, and describe the genomic characterization of five plasmids harbored by them. The results of this study will facilitate a greater understanding of the survival and persistence of such foodborne pathogens within these “built- environments”.

## Methods

### Bacterial strains and DNA isolation

*Cronobacter sakazakii* H322 and GK1025B were grown in 5 ml of Trypticase Soy Broth (TSB, BBL, Becton Dickinson, Franklin Lakes, NJ, USA) supplemented with 1% NaCl (TSBS), and incubated at 37 °C for 18 h with shaking conditions of 160 rpm (Thomas Scientific, Inc., Swedesboro, NJ, USA). Isolation of genomic DNA was performed using a 2 ml aliquot of each culture using the robotic QIACube workstation and the automated Qiagen DNeasy technology (Qiagen, Inc., Germantown, MD, USA) following the manufacturer’s recommendations as described by Jang et al*.* [17, 18].

### Whole genome sequencing, assembly, and annotation:

The single-molecule real-time (SMRT) sequel sequencing technology [19] from PacBio (Pacific Biosciences, Menlo Park, CA, USA) was utilized to create high-quality long-read datasets of *C. sakazakii* strains H322 (SRR8305966) and GK1025B (SRR8305970). The initial processing of long-sequencing reads was carried out using the RS_HGAP_Assembly.2 protocol (default parameters) implemented in the Pacific Biosciences SMRT analysis portal (version 2.3.1). Quality filtering was performed automatically during assembly using the SMRT Portal P-filter module and using the Hierarchical Genome Assembly Process 3 (HGAP3) pipeline. For generating complete genomes, a hybrid assembly strategy with UniCycler assembly software [20] implemented on the Pathosystems Resource Integration Center (PATRIC) database web-server (https://patricbrc.org/app/Assembly2). Long-read short read archive (SRA) files from PacBio and corresponding WGS datasets of the strains obtained from sequencing runs performed on an Illumina MiSeq platform (Illumina, San Diego, CA, USA) [15, 16, 29] were combined following the instructions on the web-server. The Prokaryotic Genome Annotation Pipeline (PGAP) annotations [21] of these completed genomes, plasmid sequences, and their accession numbers were released under FDA GenomeTrakr Bioproject on NCBI (PRJNA258403), which is part of FDA’s foodborne pathogen research comprehensive Bioproject at NCBI (PRJNA186875). The RAST Seedviewer was used to help provide consistent and accurate genome annotations across the genomes and plasmid sequences [22].

### Genomic analyses

The PROKSEE server (https://beta.proksee.ca/projects) was used to generate high-quality navigable maps of each circular plasmid as previously described [23]. Each *Cronobacter* plasmid’s sequence was submitted to CGE’s Plasmidfinder (https://cge.cbs.dtu.dk/services/PlasmidFinder/) for in silico determination of incompatibility plasmids such as IncF, IncHI1, IncHI2, IncN, and IncI1 plasmids [24]. For prophage sequence identification, *C. sakazakii* strain FASTA data sets were uploaded to the PHASTER (PHAge Search Tool Enhanced Release) web server and pipeline (https://phaster.ca/, last accessed 8.25.2021, [25, 26]). Mauve, Progressive Mauve, and Geneious suite 12.0 ([27]; https://www.geneious.com/) were used for alignment and visualization as needed. BLAST analysis for the presence of pH322_1 was performed on an in-house database of 683 genomes consisting of GenomeTrakr datasets along with publicly available genomes hosted at NCBI.

## Results and discussion

### Genome and plasmid characterization

The characteristics of the completed genomes and closed plasmids harbored by *C. sakazakii* strains H322 and GK1025B are summarized in Table 1. Each genome consisted of a single circular chromosome of 4,350,614 bp and 4,362,605 bp in size, contained a GC content of 56.7% and 56.9%, and 4,146 and 3,693 coding DNA sequences (CDS), respectively. Two plasmids were identified as being harbored by *C. sakazakii* H322 and three plasmids were identified to be carried by *C. sakazakii* GK1025B. None of the five plasmids identified by genome sequencing were predicted on CGE's Plasmidfinder [24]. In addition to the closed plasmids generated from long-read sequencing, PHASTER analysis showed that *C. sakazakii* strain H322 hosted four intact prophage sequences (Additional file 2: Table S2) that were located on the chromosome which included a 47.4 Kbp *Salmonella* SEN34 (NCBI accession #: NC_028699), a 37.4 Kbp *Enterobacteria* mEp235 (NC_019708), a 43.5 Kbp *Salmonella* 118970_sal3 (NC_031940), and a 17.7 Kbp *Enterobacteria* P1 (NC_005856), prophage. An incomplete generalized transducing *Salmonella* bacteriophage ES18 prophage (NC_006949) prophage was also identified. Three intact *Cronobacter* prophage sequences were additionally identified by PHASTER analysis on the chromosome of GK1025B: *Cronobacter* ENT47670 (NC_019927), *Cronobacter* ESSI_2 (NC_047854), and *Cronobacter* phiES15 (NC_018454). The complete chromosomal sequences of the two *C. sakazakii* allowed for detailed annotation and identification of mobilome sequences that could be applied for comparative analysis with other strains of *Cronobacter* and related organisms.

**Table 1:** Characteristics of *C. sakazakii* H322 and GK1025B complete genomes and plasmids from Bioproject PRJNA258403^a^

Strain ID/Plasmid	Genome/plasmid size (bp)	%GC content	Number of CDS	CRISPR arrays	NCBI biosample ID	NCBI genbank ID	NCBI accession number
H322	4,350,614	56.7	4146	1	SAMN06124518	CP078110	MRXM01000000
pH322_1	100,741	50.2	137			CP078111	
pH322_2	118,185	56.8	118			CP078112	
GK1025B	4,362,605	56.9	3693	2	SAMN04329637	CP078106	MCOE01000000
pGK1025B_1	101,769	51.1	141			CP078107	
pGK1025B_2	120,182	56.6	133			CP078108	
pGK1025B_3	46,5 28	51.0	82			CP078109	

^a^Information was obtained from NCBI (https://www.ncbi.nlm.nih.gov/genome/browse/#!/prokaryotes/1170/) and summarized

### Description of H322 plasmids: pH322_1 and pH322_2

**Fig. 1:**
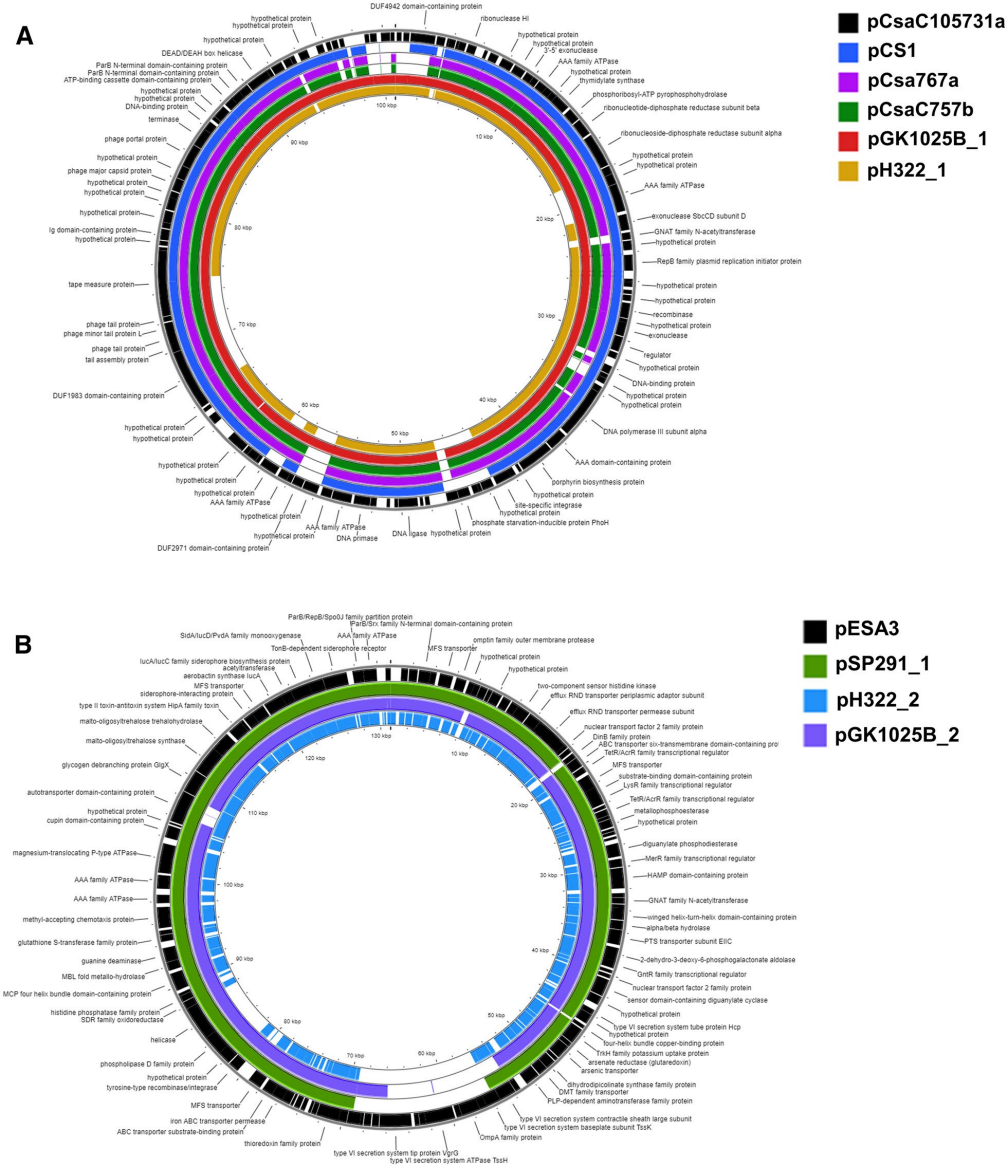
**A** Sequence alignment of *C. sakazakii* phage-plasmid class members, pH322_1 and GK1025B_1: Annotated genomes of known phage-plasmids were aligned and compared to identify conserved and divergent sequence features. The annotation of each gene is from NCBI. The inner circle represents the sequence clockwise and the scale marks indicate positions of annotated genes. GenBank annotations of the reference pCsaC105731a (100,874 bp, coding DNA sequence (CDS) in black arranged outside ring), pCS1 (110,093 pb, Blue), pCsa767a (109,716 bp, purple), pCsaC757b (109,716 bp, Green), pGK1025B_1 (101,769 bp, Red) and pH322_1 (100,741 bp, Tan) was downloaded as GFF files for the analysis using the default configuration on the PROKSEE server. Across the circular genomes, selected genes or regions of interest are shown as follows: Missing regions identified by the BLAST analysis on the CGView server’s PROKSEE software are shown as ‘gaps’ on each of the circular genomes. The analysis was carried out on PROKSEE Server from the Stothard Research Group (University of Alberta, Canada) that uses BLAST analysis to illustrate conserved and missing genomic sequences (available online: https://beta.proksee.ca/tools). **B** Sequence Comparison of *C. sakazakii* virulence plasmid class members pH322_2 and pGK1025B_2: Annotated genomes of some known virulence plasmids were aligned and compared to identify conserved and divergent sequence features. The annotation of each gene is from NCBI. The inner circle represents the sequence clockwise and the scale marks indicate positions of annotated genes. GenBank annotations of the reference pESA3 (131,196 bp, CDS in black arranged outside ring), pSP291_1 (118,136 bp, Green), pH322_2 (118,185 bp, light blue), and pGK1025B_2 (120,182 bp, purple) was downloaded as GFF files for the analysis using the default configuration on the PROKSEE server. Across the circular genomes, selected genes or regions of interest are shown as follows: Franco et al*.* [31]*,* adapted siderophore loci with Cronobactin gene, Iron ABC transporter genes, (T6SS), *parAB* genes, and the *cpa* gene. Missing regions identified by the BLAST analysis on the CGView server’s PROKSEE software are shown as ‘gaps’ on each of the circular genomes. The analysis was on the PROKSEE Server from the Stothard Research Group (University of Alberta, Canada) that uses BLAST analysis to illustrate conserved and missing genomic sequences (available online: https://beta.proksee.ca/tools)

A 100,741 bp pCS1-like closed plasmid, named pH322_1 was identified to be similar to the plasmid pseudomolecule initially predicted from H322 draft whole genome contig sequences by Chase et al*.* [15]. The sequence relatedness of pH322_1 to pCS1 harbored by *C. sakazakii* NCIMB 8272 (alias NCTC 8155) after PROKSEE analysis using the β-version of CGView Server [23] is shown in Fig. 1A. It had a GC content of 50.2% and harbored 137 CDS. Unique features contained on this plasmid included 12 mobile genetic elements comprising six copies of an Insertion Sequence 3 family transposase, three exonucleases (3–5′ exonuclease, *SbcCD* subunit D, and an unnamed exonuclease), a RecA recombinase, and a site-specific integrase (Additional file 1: Table S1). PGAP analysis also identified several phage-related genes on pH322_1 such as genes encoding for a phage exonuclease, and a phage tail fiber identified as side tail fiber protein, Stf (Additional file 1: Table S1). PHASTER analysis identified and confirmed the presence of an intact 96.9 Kb *Salmonella* SSU5 (NCBI accession number: NC_018843) prophage gene cluster [25] (Additional file 2: Table S2) located on pH322_1.

The second plasmid named pH322_2 and harbored by *C. sakazakii* H322 was 118,185 bp in size and contained a GC content of 56.8%. There were 118 CDS identified by PGAP annotation. Analysis using PROKSEE [23], showed the plasmid to be closely related to *C. sakazakii* virulence plasmid pSP291_1 harbored by ST4 *C. sakazakii* SP291 as described by Power et al*.* [30]. BLAST analysis showed that the virulence plasmid pH322_2 shares significant homology with the virulence plasmid backbones of pSP291_1 and pESA3 (data not shown). They share conserved features like the origin of replication gene, *repA*, two iron acquisition systems, an aerobactin-like siderophore (named Cronobactin, *iucABCD/iutA*), and an ABC ferric-iron transporter gene cluster (*eitCBAD*) as described by Franco et al*.* [31].

### Description of GK1025B plasmids: pGK1025B_1, pGK1025B_2 and pGK1025B_3

Complete sequences of three plasmids, pGK1025B_1, pGK1025B_2, and pGK1025B_3 was obtained by long-read sequencing and PGAP annotation (Additional file 1: Table S1 [21]). pGK1025B_1 was 101,769 bp in size and contained 141 CDS, of which 70 genes encoded for hypothetical proteins (Additional file 1: Table S1) and had a GC content of 51.1%. An intact 99.4 Kbp gene cluster encoding for a *Salmonella* SSU5 prophage (NC_018843) was identified using PHASTER and was like pH322_1 (Additional file 2: Table S2) described earlier. This is a slightly smaller sized prophage SSU5 gene cluster than what was reported by Kim et al*.* (103 Kbp; 2012) but is slightly larger than the prophage gene cluster present in pH322_1 (96.9 Kb). As was the case for pH322_1, the prophage gene cluster contained genes encoding for prophage structural proteins including terminase, capsid and tail proteins. Genes encoding for a lysin, an integrase, and a recombinase protein, and possessed a GC content of 51.1% were also noted.

pGK1025B_2 was identified as a slightly smaller version (120,182 bp) of the virulence plasmid, pESA3 (131,196 bp) that Franco et al*.* [31] described for *C. sakazakii* strain BAA-894. It contained 133 CDS, possessed a GC content of 56.6%, and harbored a homolog of *repA*, the plasmid’s origin of replication gene and a homolog of *Cronobacter* plasminogen activator, *cpa* (location: 6338 to 7276 bp). As described earlier, other noted genetic features among these virulence plasmids include: a siderophore aerobactin biosynthesis gene cluster (now named Cronobactin/siderophore receptor, *iucABCD/iutA*), a bicistronic toxin-antitoxin gene complex encoding for HigB/HipA, and a methyl-accepting chemotaxis protein I (serine chemoreceptor protein) gene.

pGK1025B_3 is a plasmid of 46,528 bp in size. It possesses a GC content of 51.1% and harbored 50 genes encoding for hypothetical proteins; however, it is a conjugative plasmid like pESA2 which is harbored by *C. sakazakii* BAA-894 [31, 33]. A ~ 16.4 Kbp type 4 secretion system gene cluster was found, and most notably it contains within this gene cluster a copy of a phospholipase D gene (*plD,* located between ~ 9308 to ~ 9841 bp, NCBI Locus Tag: AUM97_022060).

### Comparative genomic analysis of the novel phage-plasmids pH322_1 and pGK1025B_1

**Fig. 2:**
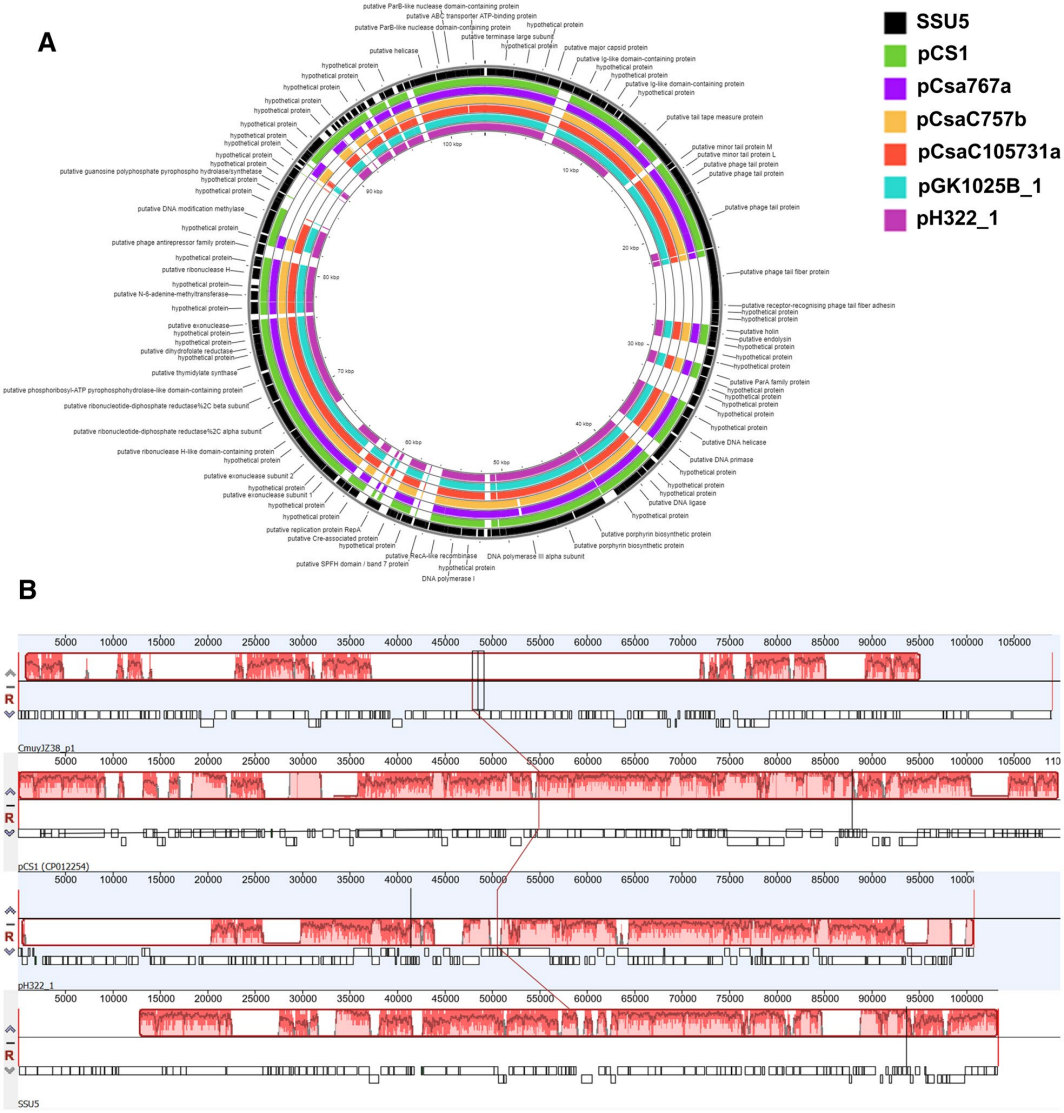
**A** Comparison of SSU5 prophage features with known *C. sakazakii* phage-plasmid class members: Four known and two new plasmid sequences from this study were compared using PROKSEE with the annotations of the *Salmonella* prophage SSU5. The inner circle represents the sequence clockwise and the scale marks indicate positions of annotated genes. GenBank annotations of the reference phage-plasmid SSU5 (CDS in Black colored ring, arranged outside ring), pCS1 (Green), pCsa767a (purple), pCsaC757b (Tan), pCsaC105731a (Red), pGK1025B_1 (Teal) and pH322_1 (Mauve) were downloaded as GFF files for analysis using the default configuration on the PROKSEE server. Across the circular genomes, selected genes or regions of interest are shown as follows: Missing regions identified by the BLAST analysis on the CGView server’s PROKSEE software are shown as ‘gaps’ (white color) on each of the circular genomes. These plasmids contained a near-complete SSU5 phage. A BLAST analysis of 630 + WGS assemblies of *Cronobacter* revealed varied coverage of the phage sequences in many plasmids (See Additional file 3: Table S3). The analysis was carried out on the PROKSEE Server from the Stothard Research Group (University of Alberta, CA) that uses BLAST analysis to illustrate conserved and missing genomic sequences (available online: https://beta.proksee.ca/tools). **B** Mauve alignment of SSU5 illustrates variations in lengths of the phage-sequences in *Cronobacter* plasmids: Plasmids from *C. sakazakii* and *C. muytjensii* were compared using the Mauve progressive alignment tool (http://darlinglab.org/mauve/user-guide/progressivemauve.html, [27, 28]) implemented on Geneious suite 12. pCS1 from *C. sakazakii* NCTC 8155 was seen to be the largest plasmid with almost 110 kb when compared to pCmuyZ38_1 from *C. muytjensii* JZ38 and the two new plasmids from this study. A detailed analysis of these plasmids, and their inclusion in plasmid-finding pipelines, would enable the identification of SSU5-like sequences from the growing number of Cronobacter WGS datasets

Initial sequence analyses described above suggested that pGK1025B_1 and pH322_1 belong to a unique category of plasmids called phage-plasmids (extrachromosomal DNA molecules that host intact prophage sequences and strictly behave like plasmids) that have been known since the 1960s [34, 35]. Sequence comparison of *repA* gene sequences from pH322_1 and pGK1025B_1 (locus_tags BTK77_021130 and AUM97_021275, respectively) suggested that these plasmids possessed a mutually exclusive origin of replication and different sub-groups of IncF1B category. It was reported that SSU5 phage bearing plasmids usually belong to IncF1B incompatibility group in other *Enterobactereaceae* members [35]. Some previously reported *Cronobacter* plasmids belong to this rare category of phage-plasmids along with the two plasmids identified in this study suggesting an expansion of genetic diversity among this emerging foodborne pathogen [15, 36]. We identified sequences containing significant homology to pGK1025B_1 and pH322_1 from among the known *Cronobacter* plasmids by BLAST analysis. The properties of these prophages are summarized in Additional file 2: Table S2 and suggest a prevalence of phage-plasmid like sequences of varied lengths in different plasmids containing homologous sequences to SSU5. Figure 2A shows the prophage gene cluster region from SSU5 in comparison with a few selected related *C. sakazakii* plasmids like pH322_1, pGK1025B-1, pCS1, pCsaC105731a, pCsa767a, and pCsaC757b. PHASTER analysis also identified and confirmed an intact *Salmonella* SSU5 prophage in the *C. muytjensii* plasmid pCmuyJZ38_1 [36] and a *E. coli* P1-like prophage on p505108-MDR and pGW1 (Additional file 2: Table S2). Analysis using the Mauve progressiveAlignment tool [28] revealed the variations in the size of the prophage region in these plasmids when compared with SSU5 as reported by Kim et al*.* [37] (Fig. 2B). To understand the distribution and prevalence of the phage-plasmids within *Cronobacter* species, we performed a BLAST analysis on 683 draft WGS genomes and closed plasmids representative of all seven species using the genes found on pH322_1. The results identified the phage-plasmid like sequences (from pH332_1) in three *C. malonaticus* strains (two ST129 and a single ST7), and 133 *C. sakazakii* strains which represented 18 different STs and these results are shown in Additional file 3: Table S3.

As noted above, the acquisition of plasmids containing prophages was a unique finding which was initially reported by Ikeda and Tomizawa for prophage P1 in *Escherichia coli* [34]. It was reported that rather than integrating its prophage genome into the host bacterium’s chromosome, its DNA was found to replicate as a circular plasmid in the lysogen. Prophage SSU5 is like *E. coli* prophages P1 and D6, which are also harbored on plasmids, are common in the Enterobacteriales, and were also among the first prophages found to be associated with plasmids [38]. Plasmids and prophages are key contributors to bacterial evolution and when found together as a single unit are often now referred to as phage–plasmids, which possess properties of both plasmids and prophages, for example, P1, N15 or SSU5. Biological characterization of these phage-plasmids is poorly understood. Pfeifer et al*.* screened over 2500 phages and 12,000 plasmids from across a diverse collection of bacterial phyla and identified 780 phage–plasmids grouped into eight distinct categories based on sequence features. This study further suggested a role for the phage-plasmids in genetically connecting phages and other mobile (and transducing) genetic elements [35]. *Salmonella* prophage SSU5 represents a different type of lysogenic phage with a circular phage-plasmid that is also very common in other members of the *Enterobacteriales*; however, they are only very rarely annotated as being phage related, much less as prophages [38]. Often a genome may contain an integration hot spot such as that found for *Lactococcus lactis* subsp. *cremoris* which contains 20% of its genome as IS elements [39]. This suggests that a genome can exist in an active evolutionary state, as it can readily accommodate new DNA and/or loose genome regions as well. Similarly, pH322_1 and pGK1025B_1 with an abundance of mobile genetic elements found in these phage-plasmid sequences (Additional file 1: Table S1) may represent such a genetic element. Furthermore, the fact that *C. sakazakii* strains H322 and GK1025B contain multiple plasmids may offer selective advantages to a bacterial host which may also reflect their adaptative abilities to persist within the nutrient-rich environment of a built environment, such as that of powdered infant formula manufacturing facilities [40].

### Genomic analysis of the virulence plasmids, pH322_2 and pGK1025B_2

The shared genome backbone (Additional file 1: Table S1) of pH322_2 and pGK1025B_2 with that of virulence plasmids, pESA3 [31] and pSP291_1 [30] is shown in Fig. 1B. Both plasmids harbored a *Cronobacter* plasminogen activator (*cpa*) encoding Protease VII or Omptin precursor (EC 3.4.23.49) (Additional file 1: Table S1) homologous to the *Salmonella* outer membrane protease, PgtE [1, 31, 32]. Both the plasmids contained a truncated ~ 13 kbp type six secretion system (T6SS) gene cluster which shares homology with a similar region harbored by pESA3, and pSP291_1. PROKSEE analysis showed that a similar truncated T6SS with a large deletion in the region for SP291_1 and pH322_2 compared to that of pESA3. In addition to the deletion within the T6SS gene cluster, pH322_2 also had a second large deletion of ~ 6 Kbp, which includes genes for a tyrosine-type recombinase/integrase, a hypothetical protein, and a phospholipase D. These results correlate with those reported by Franco et al*.* [31]; Tall et al*.* [32], Chase et al*.* [15], and Jang et al*.* [1] who had described the presence of a virulence plasmid like pH322_2, pGK1025B_2, pESA3 and pSP291_1 in a high percentage of *C. sakazakii* strains (629 of 652, 96%). Two functional T6SS clusters were reported by Wang et al., 2018 in the *C. sakazakii* strain ATCC12868 although the genome sequences are not available on NCBI for comparison [42]. In contrast, truncated T6SS segments on pESA3-like virulence plasmids reported by Franco et al*.* [31] and others had not been characterized in vivo or share sequence homology with the chromosomal clusters rendering their use just as a possible ‘signature sequence’ for this category of plasmids. A Cobalt ABC transporter gene cluster encoding for an ATP-binding protein (CbtL), permease protein (CbtK), and two copies of a substrate-binding protein gene (CbtJ) were found on pGK1025B_2, but not on pH322_2 Additional file 4: Figure S1.

### Sequence analysis of conjugative plasmid pGK1025B_3 compared with pESA2 and other *Enterobacteriaceae* plasmids

**Fig. 3:**
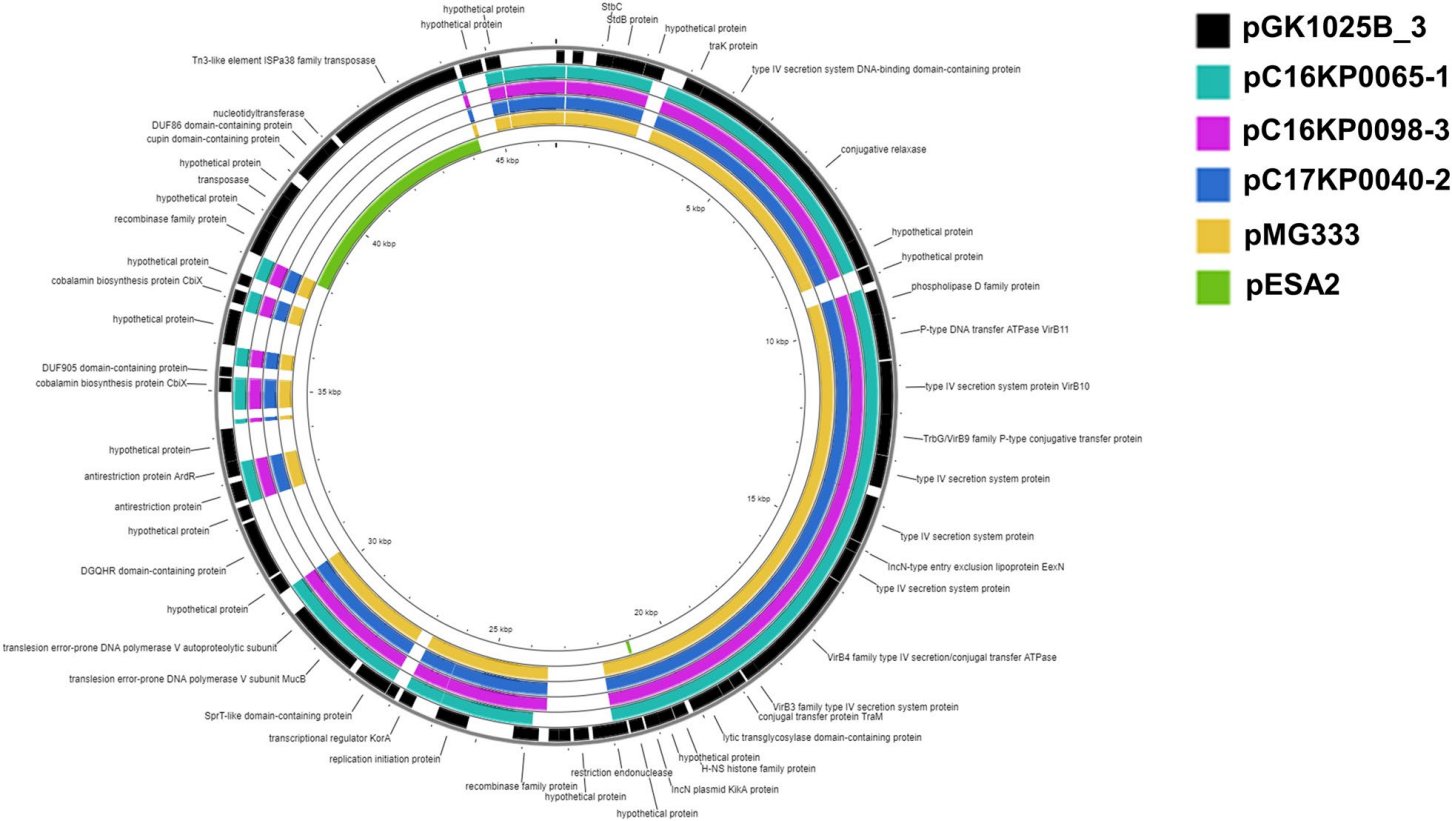
Sequence analysis of newly described *C. sakazakii* conjugative class member pGK1025B_3: Annotated genomes from pGK1025B_3 and other *Cronobacter* conjugative plasmids were compared using PROKSEE for identifying conserved and unique sequence features. The inner circle represents the sequence clockwise and the scale marks indicate positions of annotated genes. GenBank annotations of the reference pGK1025B_3 (46,528 bp, CDS in Black arranged outside ring), pC16KP0065-1 (168,415 bp, Teal), pC16KP0098-3 (49,279 bp, Purple), pC17KP0040-2 (56,619 bp, Dark Blue), pMG333 (134,435 bp, Tan), and pESA2 (31,208 bp, Green) were downloaded as a GFF file for analysis using the default configuration on the PROKSEE server. Across the circular genomes, selected genes or regions of interest are shown as follows: Missing regions identified by the BLAST analysis on the CGView server’s PROKSEE software are shown as ‘gaps’ on each of the circular genomes. The analysis was carried out on the PROKSEE Server from the Stothard Research Group (University of Alberta, CA) that uses BLAST analysis to illustrate conserved and missing genomic sequences (available online: https://beta.proksee.ca/tools)

**Fig. 4:**
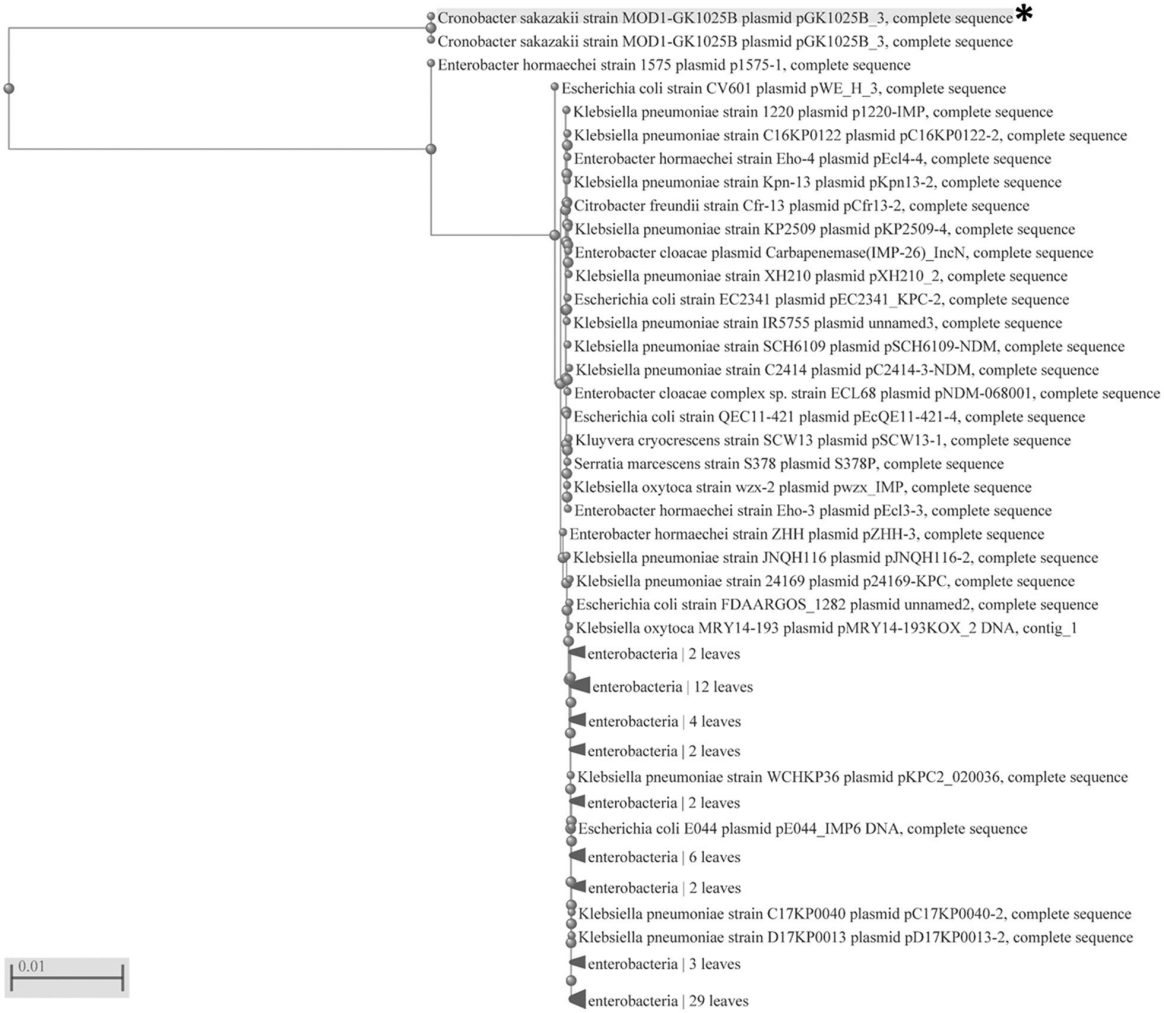
Comparative analysis of *rep* gene sequences identifies plasmid pGK1025B_3 as a unique category of conjugative plasmid in *Enterobacteriaceae*: A nearest neighbor joining phylogenetically tree was developed using a BLASTn analysis with the *rep* gene (gene locus CP078109) from *Cronobacter sakazakii* strain MOD1-GK1025B plasmid pGK1025B_3, complete sequence. This *rep* gene from this plasmid grouped separately and uniquely when compared to rest of the *rep* gene sequences from the other microorganisms. The top cluster contains the query (shown with an asterisk) and the same sequence as the unique hit. The parameters used to develop the tree included the *rep* (CP078109.1) gene from pGK1025B_3 in a BLASTn analysis against a database represented by *Enterobacteriaceae* and related endosymbionts (NCBI taxid:91347) using NCBI’s nearest neighbor algorithm. Bar marker represents 0.01 bp substitutions

Sequence alignment of newly described pGK1025B_3 compared with other conjugative class members produced on the PROKSEE server (Fig. 3) suggest that this plasmid represents a new conjugative plasmid that only has marginal sequence homology with pEAS2 from *C. sakazakii* BAA-894 [1, 33]. Interestingly, a known virulence gene coding a phospholipase D (PLD) was identified within a complete T4SS cluster harbored on pGK1025B_3 (Additional file 5: Figure S2). Results of a BLASTn analysis using the *rep* (CP078109.1) gene from pGK1025B_3 queried against *Enterobacteriaceae* and related endosymbionts (NCBI taxid:91347), showed a shared homology with many related *rep* genes. Alignment of these gene sequences, shown in Fig. 4 revealed that the *rep* gene of pGK1025B_3 clustered distinctly separated from a larger cluster of 91 *rep* genes of related plasmids of members of the *Enterobacteriaceae*. These results suggest that the *rep* gene of pGK1025B_3 may represent a novel *Cronobacter* origin of replication gene carried by a previously uncharacterized *Cronobacter* conjugative plasmid that harbors within its gene cluster a phospholipase D gene. Future surveillance studies to identify the prevalence of pGK1025B_3 like plasmids as well as functional genetic studies are needed. Phospholipase D (PLD) represents a heterogeneous group of lipolytic esterases, which are either secreted into the extracellular milieu, or directly injected into the host cell cytosol by a wide variety of Gram-positive and Gram-negative bacteria through Type 6 and Type 4 secretion systems [41]. It plays an important role in several host–pathogen physiological interactions involved in bacterial pathogenesis, including cell invasion, evasion of the host immune response through escape of or maturation avoidance within phagosomes, establishment of tissue colonization, and systemic spread. The contribution of the *Cronobacter* version of PLD in the pathogenicity of this organism needs to be further studied.

## Conclusions

The mechanisms related to the persistence of *Cronobacter* strains within the built environment such as that of powdered infant formula manufacturing facilities are currently unknown. The use of whole-genome sequencing of *Cronobacter* isolates obtained from the “built environment” as part of a routine surveillance strategy is only in its infancy but is a first step in determining the relationships of *Cronobacter* species that possess a long-term persistence phenotype in food manufacturing facilities. WGS analyses demonstrated that these two persistent *C. sakazakii* strains possess five plasmids of which fall into three different plasmid classes, such as the virulence plasmids pSP291_1 and pESA3 originally characterized by Power et al*.* [30] and Franco et al*.* [31], a prophage bearing pCS1-like plasmid originally described by Chase et al*.* [15], and an uncharacterized conjugative plasmid like that of pGK1025B_3 that possesses a phospholipase D gene within its T4SS gene cluster. The genomic information about these two highly persistent *C. sakazakii* strains H322 and GK1025B provides insights to design further in-depth investigations of a facility’s microbiota profile. This information could also be used in future studies to develop basic differences between non-pathogenic and pathogenic microorganisms found within these food manufacturing environments. Finally, future analysis of the genome sequences of wild-type *C. sakazakii* strains will shed more light on the importance of plasmids and phage-plasmids and their role in survival and persistence in PIF manufacturing environments, and as causative agents of severe-invasive human infectious diseases. This study highlights the increased discriminatory power of WGS analysis and emphasizes the need for furthering extended surveillance studies and provides insights linking the genotype–phenotype of *C. sakazakii* from previously published longitudinal surveillance investigations.

## Supplementary Information

**Additional file 1:** **Table S1**. PGAP annotation of genes carried on pH322_1, pH322_2, pGK1025B_1, pGK1025B_2, and pGK1025B_3 that are harbored by C. sakazakii H322 and GK1025B.a.

**Additional file 2: Table S2**. Results of PHASTER analysis for various Cronobacter plasmids including pH322_1, pH322_2, pGK1025B_1, pGK1025B_2, and pGK1025B_3.a.

**Additional file 3: Table S3**. BLAST analysis of 683 Cronobacter genomes housed in a local database for the presnece of phage-plasmid pH322_1.

**Additional file 4: Figure S1** Multiple alignment analysis of the *Cronobacter *arsenic operon within the T6SS of virulence plasmids, pSP291_1, pH322_2, pGK1025B_2, and pESA3, as displayed by using Geneious suite. The black horizontal bar indicates the consensus sequence. The blue line indicates sequence coverage; the green represents percent identity with red presenting little homology; and green representing high homology. The arsenic operon consists of three genes: arsenate reductase (*arsC*, glutaredoxin), arsenic transporter, and a gene encoding a metalloregulator ArsR/SmtB family transcription factor. The operon is flanked by genes encoding for a TrkH family potassium uptake protein and dihydrodipicolinate synthase family protein.

**Additional file 5: Figure S2 Cronobacter** phospholipase D family protein within the T4SS of pGK1025B_3 as displayed by using Geneious suite. The phospholipase D family protein is flanked by genes encoding for two hypothetical proteins and a conjugative relaxase and *VirB11* (a member of the superfamily of traffic ATPases). Other adjacent genes include *VirB10,* which has a role in regulating substrate transfer to the extracellular space, and *VirB9* which encode for a channel protein that forms heterodimers with VirB7. VirB7 is localized at the outer membrane and plays a stabilizing role with the other VirB proteins during assembly of the T4SS pilus.

## Data Availability

Not applicable.
