# Supplementary figures and images for: Complete genome sequences and genomic characterization of five plasmids harbored by environmentally persistent Cronobacter sakazakii strains ST83 H322 and ST64 GK1025B obtained from powdered infant formula manufacturing facilities
Source: Gut Pathog. 2022 Jun 6;14:23. doi: 10.1186/s13099-022-00500-5 (PMC9169379; doi:10.1186/s13099-022-00500-5)

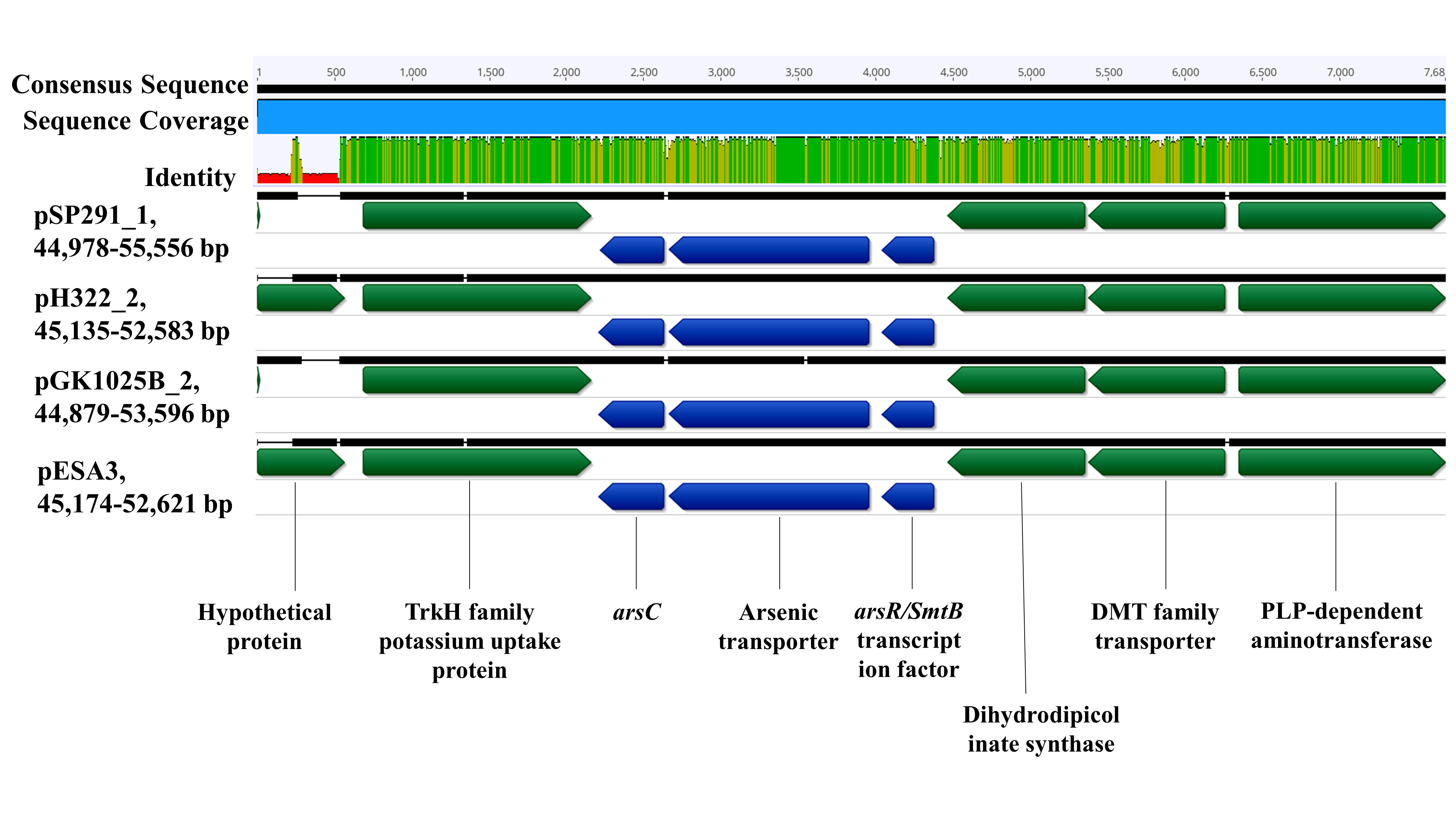

Supplement: Supplementary file 4 — Additional file 4: Figure S1 Multiple alignment analysis of the Cronobacter arsenic operon within the T6SS of virulence plasmids, pSP291_1, pH322_2, pGK1025B_2, and pESA3, as displayed by using Geneious suite. The black horizontal bar indicates the consensus sequence. The blue line indicates sequence coverage; the green represents percent identity with red presenting little homology; and green representing high homology. The arsenic operon consists of three genes: arsenate reductase (arsC, glutaredoxin), arsenic transporter, and a gene encoding a metalloregulator ArsR/SmtB family transcription factor. The operon is flanked by genes encoding for a TrkH family potassium uptake protein and dihydrodipicolinate synthase family protein. [file 13099_2022_500_MOESM4_ESM.tif]

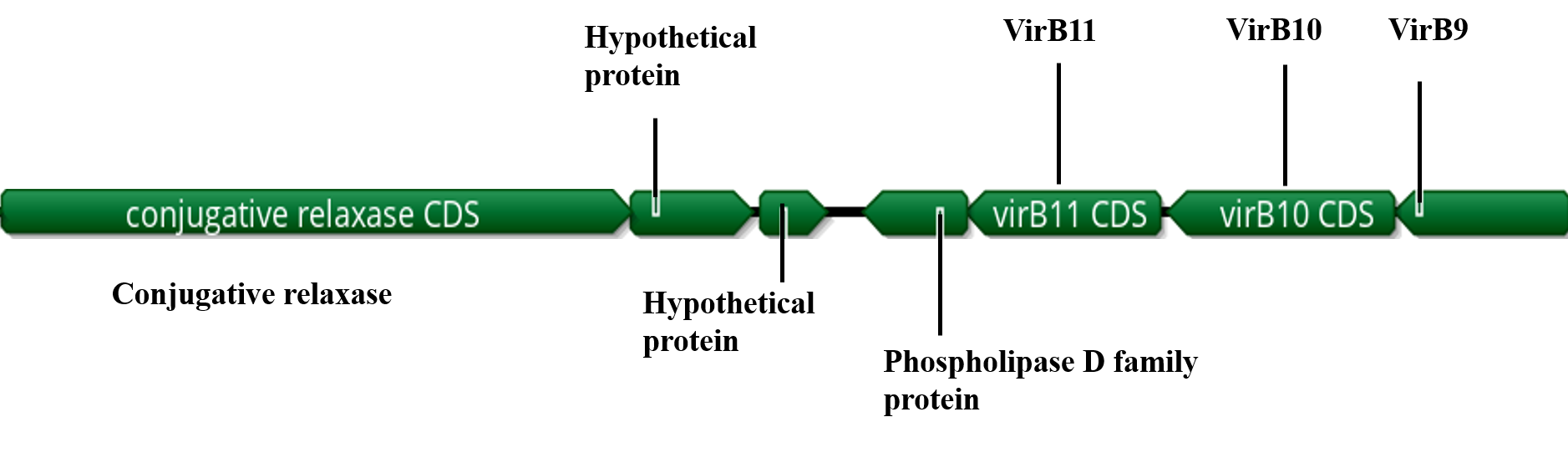

Supplement: Supplementary file 5 — Additional file 5: Figure S2 Cronobacter phospholipase D family protein within the T4SS of pGK1025B_3 as displayed by using Geneious suite. The phospholipase D family protein is flanked by genes encoding for two hypothetical proteins and a conjugative relaxase and VirB11 (a member of the superfamily of traffic ATPases). Other adjacent genes include VirB10, which has a role in regulating substrate transfer to the extracellular space, and VirB9 which encode for a channel protein that forms heterodimers with VirB7. VirB7 is localized at the outer membrane and plays a stabilizing role with the other VirB proteins during assembly of the T4SS pilus. [file 13099_2022_500_MOESM5_ESM.tif]
